# Bioactive Effect of Plasma-Rich in Growth Factors (PRGFs) on Cell-Based In Vitro Models of Skin Inflammation in Relation to Inflammatory Skin Disorders

**DOI:** 10.7759/cureus.74252

**Published:** 2024-11-22

**Authors:** Eduardo Anitua, Roberto Tierno, Zuriñe Martínez de Lagrán, Mohammad H Alkhraisat

**Affiliations:** 1 Medicine, University Institute for Regenerative Medicine and Oral Implantology (UIRMI), Vitoria, ESP; 2 Regenerative Medicine, Biotechnology Institute (BTI), Vitoria, ESP; 3 Dermatology and Pathologic Anatomy, University Hospital of Alava, Vitoria, ESP

**Keywords:** bioactivity, heat-inactivation, leukocyte exclusion, plasma rich in growth factors/ prgf, skin inflammation

## Abstract

Plasma rich in growth factors (PRGFs) has proven potentially beneficial as a bioregenerator in patients with chronic skin disorders due to its anti-inflammatory effect. However, its therapeutic potential may be limited by soluble autoimmune components associated with inflammatory dermatoses in blood plasma. To evaluate the impact of skin health status on cell bioactivity, PRGF was prepared from healthy (H) donors as well as from individuals with atopic dermatitis (AD), psoriasis (PS), or lichen sclerosus (LS). Leukocyte exclusion and heat inactivation (Immunosafe treatment) were evaluated as potential methods to reduce the inflammatory components of the samples under study. The biological effect of PRGF-derived formulations was investigated using cell-based in vitro skin inflammation models, including human dermal fibroblasts (HDFs) and human epidermal keratinocytes (HEKs) exposed to a pro-inflammatory environment. The data confirmed that viability, proliferation, and migration rates were enhanced in inflamed cell cultures supplemented with PRGF formulations compared to those maintained in standard culture media. Nevertheless, significant differences have been identified. About the healthy control, inflamed epidermal keratinocytes supplemented with most PRGF-based formulations obtained from pathological donors (PS/LS) showed lower viability. Heat inactivation significantly promoted cell proliferation in epidermal keratinocytes supplemented with SP (PS/LS) and L-PRP supernatant (LSP) samples (AD), and also cell migration in inflamed HDF (AD/H/LS) and HEK (AD/LS) models supplemented with LSP. Leukocyte exclusion improved cell behavior in terms of migration with the only exception of LSP from individuals with AD added to inflamed HEK cultures. In conclusion, PRGF derived from pathological patients contains autoimmune components that could compromise its effectiveness as a therapy for treating individuals with chronic inflammatory disorders. However, heat inactivation (Immunosafe treatment) or leukocyte exclusion could minimize local adverse effects.

## Introduction

Cutaneous and mucocutaneous inflammatory disorders associated with autoimmune diseases are a growing concern because they show a continuously increasing prevalence, may predispose to the development of other comorbidities, and often have a significantly higher incidence in the most vulnerable population groups [1]. Atopic dermatitis (AD), psoriasis (PS), and lichen sclerosus (LS) are among the most common inflammatory skin conditions, with estimated prevalences around 2.6% for AD [2], 2%-3% for PS [3], and 0.1%-0.3% for LS [4]. In the case of AD, females are more likely to suffer from AD than males. Even though its prevalence varies from 10% to 20% in the pediatric population, it is also steadily rising among older adults. In addition to the problems derived from the disease itself, predominantly characterized by eczema, dry skin, and itching, there is a predisposition to the development of other comorbidities, such as asthma, allergic rhinitis, or food allergies [5]. PS is a chronic, inflammatory, autoimmune disease characterized by erythematous plaques covered with silvery scales. Even though epidemiological data on psoriatic disease are uncertain, higher prevalence rates have been reported in countries at higher latitudes [6], and studies reporting the age‐specific prevalence of PS showed an increasing trend with age until around 60 or 70 years of age, and therefore, there is a higher incidence of PS in the elderly. Due to systemic inflammation, psoriatic disease is often associated with serious consequences on patients’ quality of life, and also with other comorbidities that negatively impact social and private life, including psoriatic arthritis, metabolic syndrome, inflammatory bowel disease, and psychological disorders. Some studies suggest that PS could shorten the lifespan of patients by 4-10 years [7]. With a higher incidence in premenarchal girls and menopausal women, LS is an underdiagnosed dermatosis characterized by skin atrophy and hypopigmentation, which typically involves anogenital areas. LS often leads to itching, pain, skin fragility, bleeding, chronic fissuring scars, and secondary infections that are strongly associated with certain types of cancer, autoimmune diseases, and poor psychosexual health [8].

Therefore, the physiological processes that regulate cutaneous anatomical integrity are severely dysregulated in patients suffering from chronic inflammatory conditions that are commonly associated with autoimmune disorders [9]. Despite the advances and benefits of biologic therapies targeting specific dysregulated immune pathways in each condition - such as interleukin-4 (IL-4), IL-13, and Janus Kinase 1 (JAK1) inhibitors in AD [10], TNF-α inhibitors, and IL-17 or IL-23 antagonists in psoriasis (PS) [11], and TNF-α inhibitors and IL-4 and IL-13 signaling blockers in LS [12] - approximately 20%-50% of patients do not experience substantial symptom improvement, even with these treatments [13,14]. Consequently, over the last few decades, advances in biotechnology, regenerative medicine, and new biomaterials have made it possible to envision a future in which this set of new disciplines will be able to slow down or modulate some of the most evident signs and symptoms of inflammatory skin conditions [15]. Platelet-rich plasma (PRP) has emerged as a promising therapeutic option in aesthetic dermatology, trichology, ophthalmology, traumatology, reproductive medicine, and wound management [16]. PRP contains a wide range of growth factors, cellular factors, cell adhesion molecules, cytokines, and chemotactic factors, which are essential for tissue regeneration, inflammation modulation, and angiogenesis. Accumulating evidence shows that bioactive morphogens present in PRP play critical roles in all three phases of wound healing and repair cascade (inflammation, proliferation, and remodeling). As concluded by Huber et al. [17], the intrinsic composition of PRP suggests that this blood-derived product can function as an immunomodulatory agent, primarily due to the diverse array of secreted cytokines, especially the role of transforming growth factor-β1 (TGF-β1) in the differentiation of T regulatory cells (Treg).

Regarding the management of the above-mentioned chronic inflammatory skin pathologies, recent research has revealed that PRP could have considerable effects in reducing inflammation and modulating the immune response [18,19]. Nevertheless, considering the autologous nature of platelet-derived products, it remains unclear to which extent PRP compositions and their biological effects are determined by the immune cell and cytokine profile of individuals [20]. After considering its clinical use, PRP from pathological patients should be characterized and optimized since the presence of soluble autoimmune components, such as immunoglobulins, complement proteins, inflammatory cytokines, or autoantibodies, in blood collected from donors with inflammatory skin conditions may impair their therapeutic potential. This study aimed to evaluate the biological effect of plasma rich in growth factors (Endoret® PRGF®, Biotechnology Institute [BTI], Vitoria, Spain) obtained from patients with different inflammatory skin conditions using a cell-based in vitro model of skin inflammation. According to some of the most widely used PRP classifications, PRGF is classified as pure-PRP, P2-x-Bβ category, and 24-00-11 code [21]. Moreover, optimization steps in the preparation protocol, including processes such as heat inactivation and leukocyte exclusion, were tested to minimize the immunogenic potential of the samples.

## Materials and methods

Preparation of PRGF-Endoret® formulations

**Figure 1:**
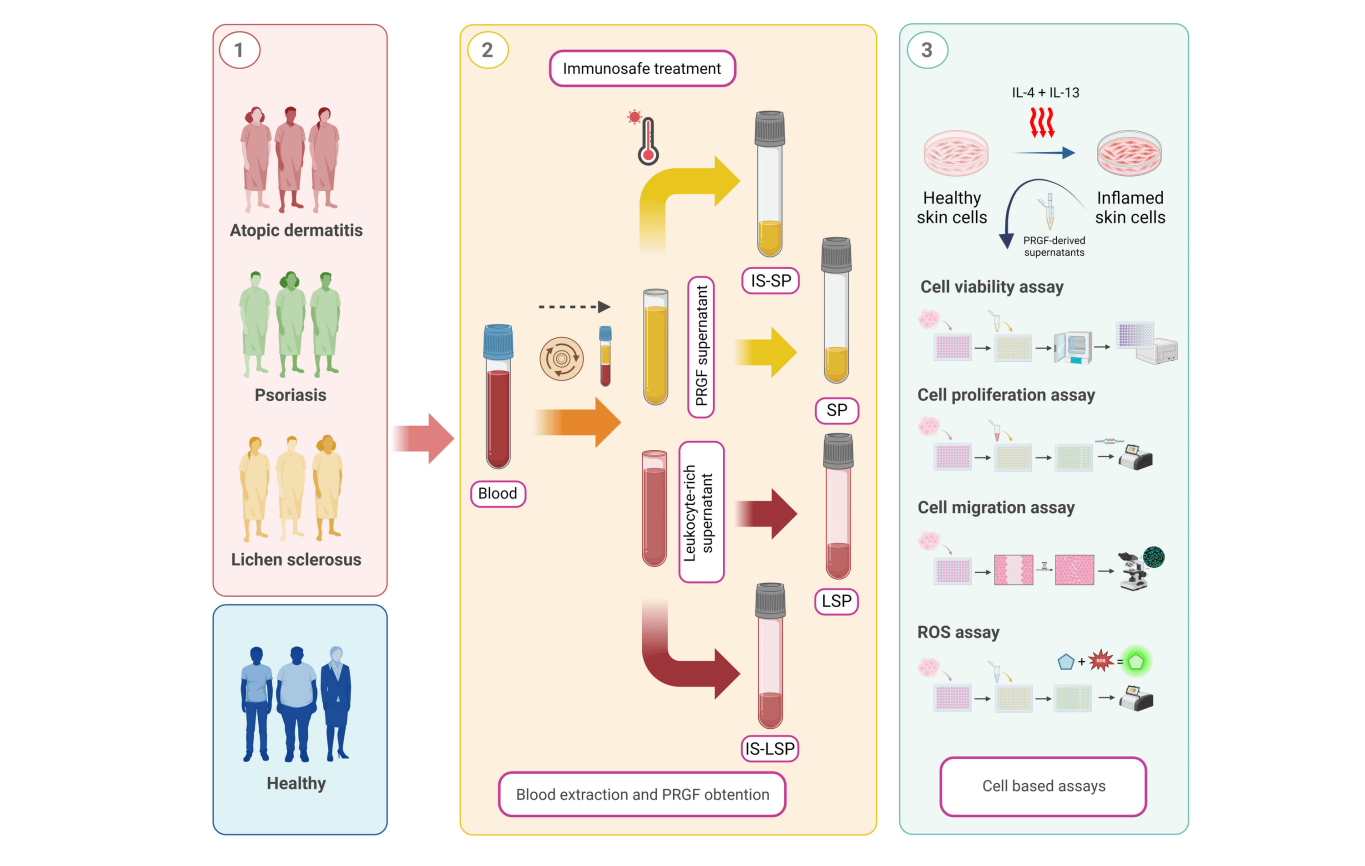
Experimental design of the study. Created with BioRender.com. LS, lichen sclerosus; PRGF, plasma rich in growth factor; LSP, L-PRP supernatant; IS-SP, PRGF Immunosafe supernatant; IS-LSP, L-PRP Immunosafe supernatant; ROS, reactive oxygen species

Figure 1 represents the global experimental and analytical pipeline developed in this study. Blood from patients with different chronic inflammatory skin disorders was extracted (*N* = 20). Three pathological groups were selected for study: PS (*n* = 5), LS (*n* = 5), and AD (*n* = 5). A supplementary group of healthy donors without any diagnosed skin conditions was also included (*n* = 5). Patients’ basic characteristics and clinical details are shown in Table 1. PRGF-Endoret® technology was used to obtain PRGF-based formulations under sterile conditions, following the guidelines provided by the manufacturer (BTI). Once written informed consent was acquired, whole blood was collected into a total of 20 tubes (9 mL) containing 0.4 mL of 3.8% (w/v) sodium citrate. Tubes were centrifuged at 580*g* for 8 minutes at room temperature using a System V centrifuge (Biotechnology Institute) to facilitate the separation of blood components based on their density gradient. From 10 of the tubes, the entire plasma column was collected while avoiding the leukocyte-rich buffy coat, and this was identified as plasma rich in growth factors (PRGF). From the remaining 10 tubes, the whole plasma column, including the buffy coat, was collected and referred to as leukocyte-rich platelet-rich plasma (L-PRP). Subsequently, half of the sample volume was activated with a 10% (m/v) solution of CaCl_2_ (20 μL/mL) and incubated for 1 hour at 37 °C in a Plasmaterm H heater (BTI). After activation, the cell-free supernatants were filtered through a 0.2 μm filter to obtain both the PRGF supernatant (SP) and the L-PRP supernatant (LSP).

**Table 1:** Patients’ basic characteristics and clinical details. unk., unknown

Patient ID	Condition	Age (years)	Sex	Current medication
H1	Healthy	33	Female	-
H2	Healthy	69	Male	-
H3	Healthy	50	Male	-
H4	Healthy	52	Female	-
H5	Healthy	51	Male	-
AD1	Atopic dermatitis	15	Male	-
AD2	Atopic dermatitis	28	Male	-
AD3	Atopic dermatitis	18	Female	Dupilumab
AD4	Atopic dermatitis	52	Female	Apremilast
AD5	Atopic dermatitis	28	Female	-
P1	Psoriasis	65	Female	Glucocorticoids
P2	Psoriasis	unk.	Male	-
P3	Psoriasis	36	Female	Glucocorticoids
P4	Psoriasis	48	Female	-
P5	Psoriasis	48	Female	-
LS1	Lichen sclerosus	unk.	Female	-
LS2	Lichen sclerosus	61	Female	Glucocorticoids
LS3	Lichen sclerosus	20	Female	Glucocorticoids
LS4	Lichen sclerosus	73	Female	Glucocorticoids
LS5	Lichen sclerosus	unk.	Female	-

The other half of the sample volume was subjected to a previously established heat inactivation procedure (Immunosafe). This method was described by Anitua et al. [22]. These samples were activated with a 10% (m/v) solution of CaCl_2_ (20 μL/mL), as previously described, and were maintained for the first 15 minutes at 37 °C, followed by 45 minutes at 56 °C in a Plasmaterm H Plus heater (BTI). Finally, the cell-free Immunosafe supernatants were filtered using a 0.2 μm filter to obtain both the PRGF Immunosafe supernatant (IS-SP) and the L-PRP Immunosafe supernatant (IS-LSP). The L-PRP samples were also treated according to the inactivation procedure to obtain the Immunosafe-treated leukocyte-rich PRP supernatant (IS-LSP). These formulations were aliquoted and stored at -80 °C until use.

Cell-based bioassays

In vitro bioactivity testing of PRGF formulations was performed using human dermal fibroblasts (HDFs; ScienCell Research Laboratories, San Diego, CA) and human epidermal keratinocytes (HEKs; ATCC, Manassas, VI). In summary, cells were maintained at 37 °C in a humidified incubator with 5% CO_2_ until they reached confluence, utilizing the appropriate culture media: fibroblast growth media-FM (InnoProt, Derio, Spain) supplemented with 100 U/mL penicillin/streptomycin (Thermo Fisher Scientific, Waltham, MA), fibroblasts growth supplement (FGS; InnoProt, Derio, Spain) and 2% v/v fetal bovine serum (Sigma-Aldrich, St. Louis, MO) for HDF, and defined keratinocyte serum-free medium (SFM; Thermo Fisher Scientific, Waltham, MA) supplemented with 5 μg/mL gentamycin (Sigma-Aldrich) for HEK. Subsequently, HDFs and HEKs were detached using an animal-origin-free trypsin-like enzyme (TrypLE Select, Gibco-Invitrogen, Grand Island, NY) and then seeded at passages 3-5 in various culture plates for different cell bioactivity assays, including the viability assay, proliferation assay, ROS assay, and migration assay.

In this study, HDF and HEK cultures for the inflamed model were synchronized in basal medium for 24 hours, while healthy control wells were maintained in complete medium during the same period. The cell seeding densities were set at 8,000 cells/cm² for proliferation assays, 15,000 cells/cm² for Reactive Oxygen Species (ROS) assays, and 20,000 cells/cm² for viability assays in 96-well culture plates. Once the cells reached confluence, the healthy control wells continued in a complete medium for an additional 24 hours. To simulate inflamed skin, IL-4 and IL-13 were added to the basal culture media of the inflamed model at a final concentration of 50 ng/mL and incubated for another 24 hours. After washing with phosphate-buffered saline (PBS), the healthy control wells were again supplied with a complete medium, while the inflamed control wells received basal cell culture media without supplements, along with the interleukin cocktail (50 ng/mL). For assessing the effects of PRGF treatments on inflamed cells, the media consisted of basal cell culture media combined with the interleukin cocktail (50 ng/mL) and 20% (v/v) of the obtained PRGF supernatants. Both control and treated cells were maintained under these conditions for 48 hours, with all assays performed in triplicate. Cell viability was assessed using the WST-1 colorimetric assay (Sigma-Aldrich) according to the manufacturer’s instructions. Relevant controls were included, such as cells cultured in complete media with 6% (v/v) dimethyl sulfoxide (DMSO; cytotoxic control), as well as positive controls (cells in normal conditions with complete media) and negative controls (cells in complete media with the interleukin cocktail). The proliferation rate, expressed as total DNA concentration, was analyzed using the fluorimetric CyQuant assay (Molecular Probes-Life Technologies, Grand Island, NY) following the manufacturer’s guidelines. Cellular ROS levels were determined using the DCFDA/H2DCFDA cellular ROS kit assay (ABCAM, Cambridge, UK) and detected via fluorescence spectroscopy as per the manufacturer’s recommendations. Similar to the cell viability assays, positive and negative controls for cell proliferation and cellular ROS were included. Since DCFDA/H2DCFDA and CyQuant have similar excitation/emission spectra, we stained cell cultures with DCFDA/H2DCFDA and CyQuant in replicated wells, and ROS per cell levels were quantified by dividing DCFDA/H2DCFDA fluorescence with CyQuant fluorescence [23].

For the migration assay, cells were seeded at a high density of 30,000 cells/cm² in migration inserts (Ibidi GmbH, Martinsried, Germany) placed on 24-well plates, and incubated with a complete medium for 48 hours. After this incubation, the inserts were carefully removed, creating two cell monolayers separated by a 500 µm gap. Following a wash with PBS, pre-treatments were applied: complete medium for the healthy control and basal cell culture media combined with 50 ng/mL of the interleukin cocktail for the inflamed model, both done in triplicate. The cells were maintained under these conditions for 24 hours. Finally, after another wash with PBS, treatments were administered as follows: complete medium was added to the healthy control wells, basal cell culture media without supplements along with the interleukin cocktail (50 ng/mL) to the inflamed controls, and a combination of basal cell culture media, the interleukin cocktail (50 ng/mL), and 20% (v/v) of the obtained PRGF supernatants to the treatment wells. After the treatments were removed, the cell nuclei were stained with Hoechst-33342 (Molecular Probes-Life Technologies). The central area of the septum was photographed before and after the treatment period using phase contrast and fluorescence imaging with a digital camera attached to an inverted microscope (Leica DFC300 FX and Leica DM IRB, Leica Microsystems). The number of migrated cells was quantified using FIJI [24], and the results were presented as the number of cells per mm² of the gap.

Statistical analysis

Statistical analyses were performed using R [25]. Generalized linear mixed-effects models were developed, incorporating donor as a random effect and analysis of variance (ANOVA) tests were performed to analyze the effect of pathological conditions and optimization steps on cell biological activity measures. The assumptions underlying parametric statistics were evaluated in the model residuals through visual inspection (QQ plots and density distributions) and significance tests (the Shapiro-Wilk test for normality and Levene’s test for homoscedasticity). Multiple comparisons were conducted using Bonferroni-corrected post hoc tests. The significance level was set at α = 0.05. The following symbols represent statistical significance: *P < 0.05 indicates significant differences between samples of each pathological condition versus its respective sample of the healthy group. #*P *< 0.05 designates significant differences for each sample after the Immunosafe treatment application. $*P *< 0.05 specifies the existence of significant differences between leukocyte-free and leukocyte-rich specific formulations.

## Results

Data regarding the hematological and serological characterization of blood, SP, and IS-SP formulations have been previously published [26]. Moreover, a detailed proteomic characterization of the studied formulations obtained from donors with different skin conditions can be also consulted in a study by Anitua et al. [27].

**Figure 2:**
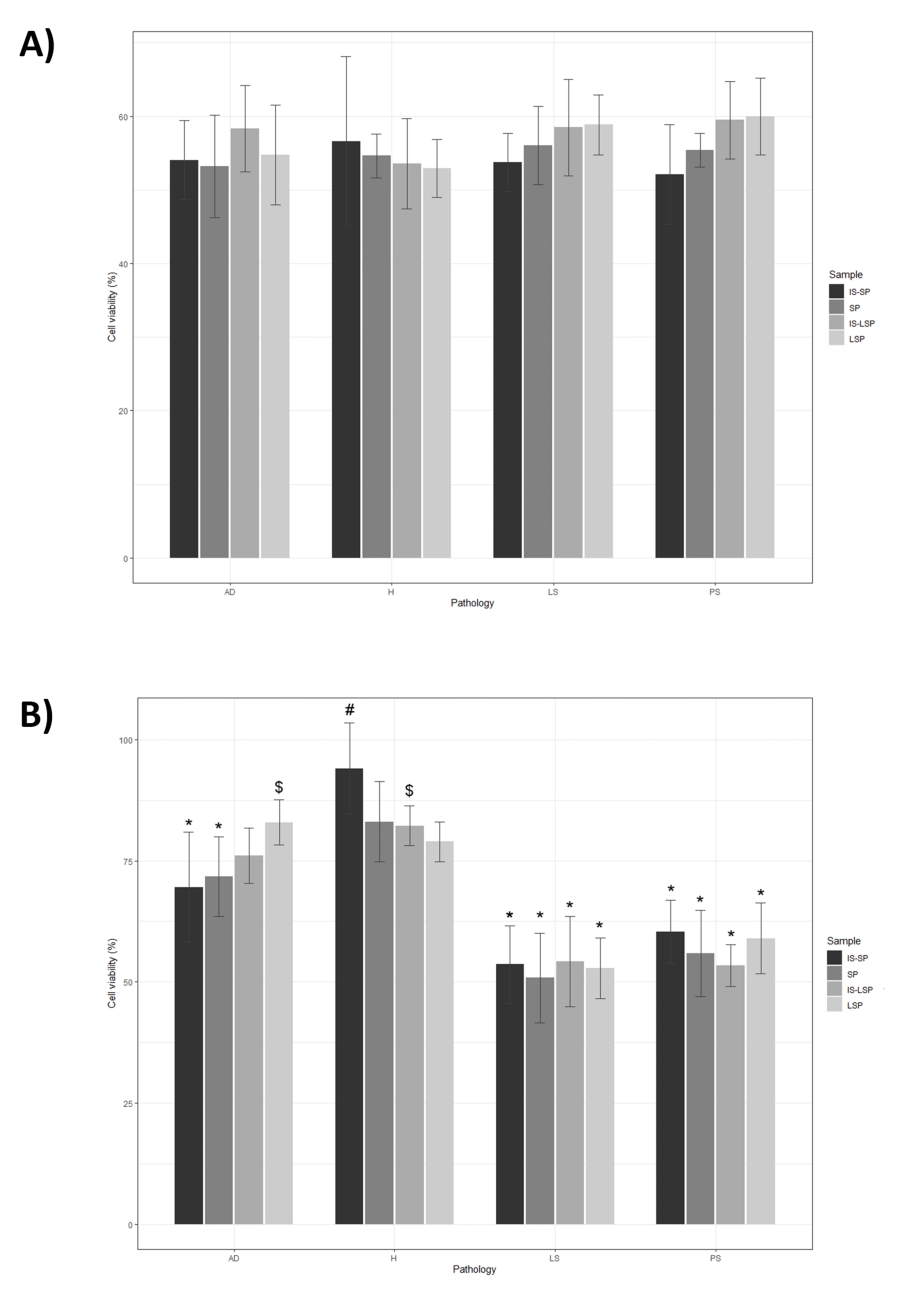
Barplots representing cell viability of inflamed in vitro models supplemented with plasma-derived formulations obtained from healthy and pathological donors using the following cell lines: (A) HDF and (B) HEK. Symbols denote statistical significance (*P *≤ 0.05): *, differences between pathological donors and healthy donors within a specific formulation; #, differences between raw formulations (SP) and those subjected to the Immunosafe thermal inactivation treatment (IS-SP); and $, differences between PRGF-derived supernatants (SP) and leukocyte-rich supernatants (LSP). PRGF, plasma rich in growth factor

**Figure 3:**
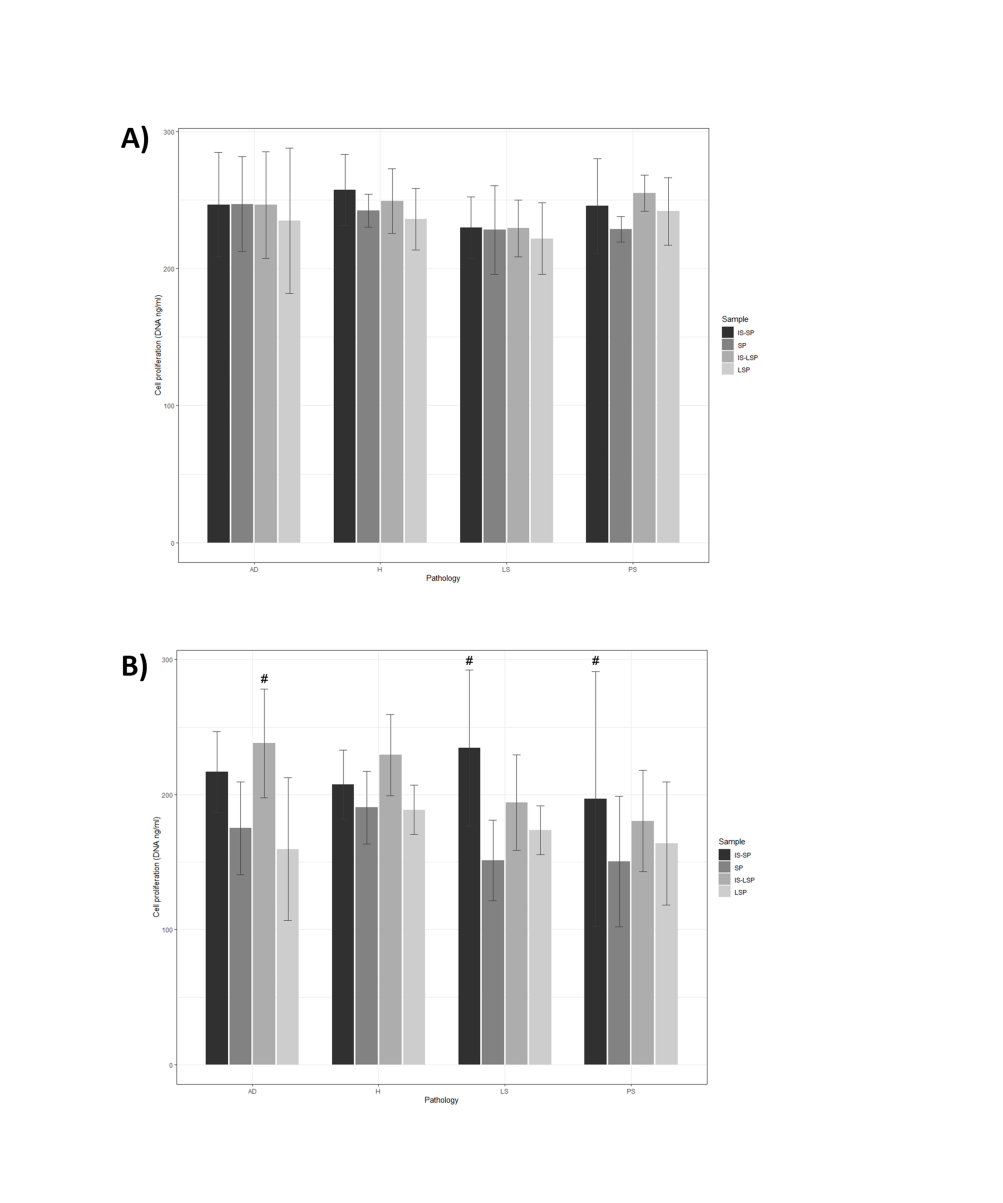
Bar plots representing cell proliferation of inflamed in vitro models supplemented with plasma-derived formulations obtained from healthy and pathological donors using the following cell lines: (A) HDF and (B) HEK. Symbols denote statistical significance (*P *≤ 0.05): *, differences between pathological donors and healthy donors within a specific formulation; #, differences between raw formulations (SP) and those subjected to the Immunosafe thermal inactivation treatment (IS-SP); and $, differences between PRGF-derived supernatants (SP) and leukocyte-rich supernatants (LSP). PRGF, plasma rich in growth factor

**Figure 4:**
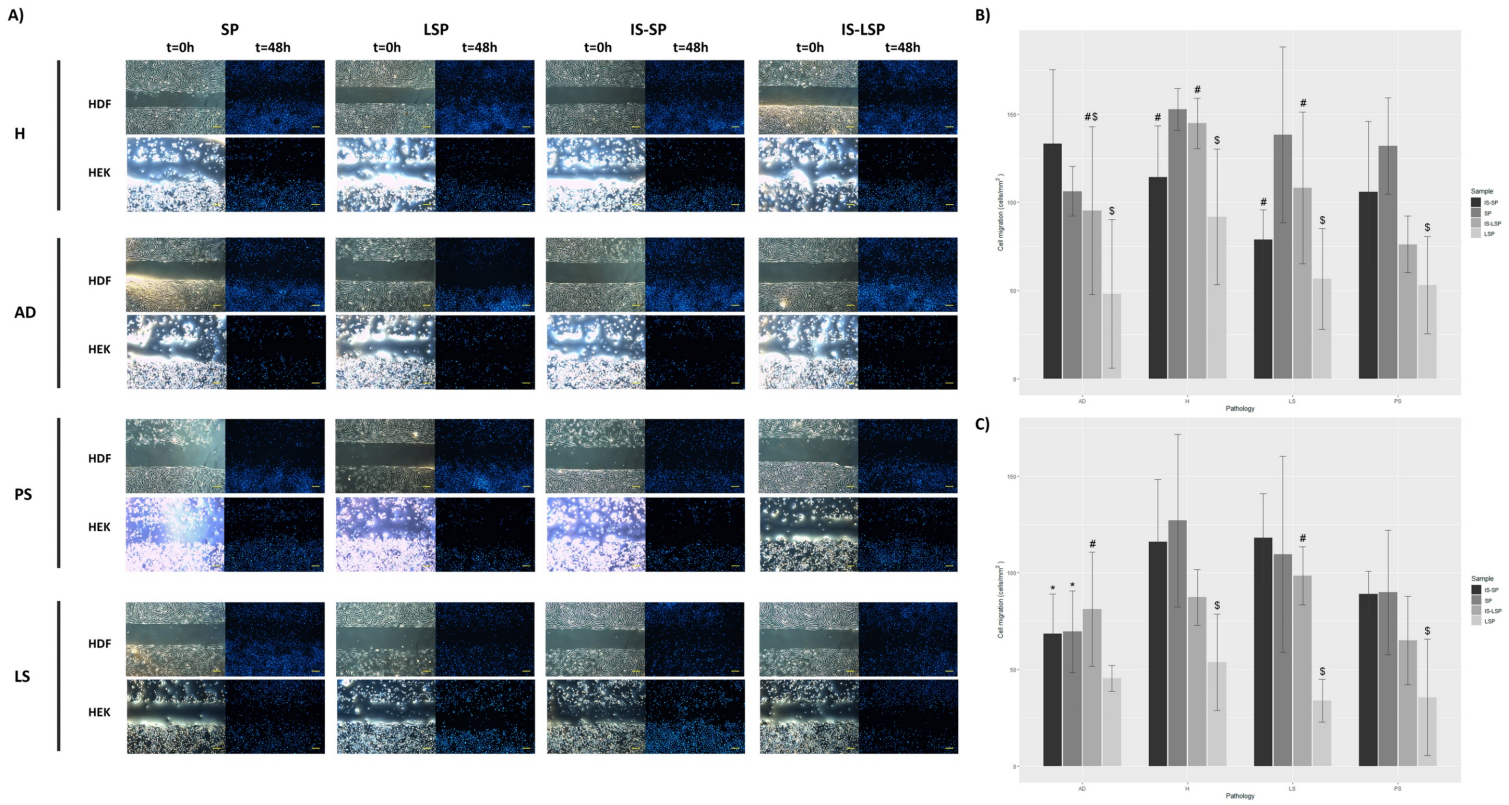
Time-lapse analyses of cell migration: (A) high-magnification, phase-contrast (t = 0 hours), and fluorescence images (t = 48 hours) showing HDF and HEK migration in response to cell culture supplementation with multiple PRGF-derived formulations obtained from healthy donors and individuals with different chronic skin disorders; (B) quantification of HDF cell migration assay, and (C) quantification of HEK migration assay. Scale bars: 25 µm. PRGF, plasma rich in growth factor; HDF, human dermal fibroblast; HEK, human epidermal keratinocyte

In the present study, HDFs and HEKs were used as an in vitro inflammation model to analyze the biological activity of obtained supernatants to mimic skin inflammatory conditions in patients with chronic cutaneous disorders. When compared to inflamed cell cultures maintained in routine culture media, all the studied formulations improved cell viability, proliferation, ROS, and migration significantly (*P *≤ 0.0001). Bar plots representing viability (Figure 2), proliferation (Figure 3), and migration (Figure 4) of inflamed HDF and HEK cells cultured in a medium supplemented with the plasma-derived formulations obtained from donors with different skin conditions are shown.

Effect of health status

When compared with the healthy control, the effect of supplementing with plasma-derived formulations obtained from pathological donors on HDF viability was not statistically significant independently of the medical situation of the donor. By contrast, considering cell viability in HEK cultures, significant differences between formulations obtained from healthy and pathological donors were identified for SP (pAD = 0.032, pPS ≤ 0.0001, and pLS ≤ 0.0001) and IS-SP (pAD ≤ 0.0001, pPS ≤ 0.0001, and pLS ≤ 0.0001), and also for LSP (pPS ≤ 0.0001 and pLS ≤ 0.0001) and IS-LSP (pPS ≤ 0.0001 and pLS ≤ 0.0001) extracted from donors with LS and PS. Regarding the effect of plasma-derived formulations on cell proliferation, no significant differences were detected between formulations from pathological individuals and those of healthy individuals in HDF cultures. On the other hand, as revealed by HDF migration assays, significant differences between raw and heat-inactivated SP and LSP formulations obtained from healthy donors (pSP = 0.040 and pLSP = 0.0053) and donors with LS (pSP=0.0019 and pLSP=0.0064) were identified. Moreover, when compared with SP and IS-SP derived from healthy donors, those obtained from donors with AD significantly reduced HEK migration (pSP = 0.0036 and pIS-SP = 0.019).

Effect of leukocyte exclusion

The presence of leukocytes in IS-SP from healthy donors significantly reduced HEK viability (*P *= 0.0092). In contrast to cell viability results, neither HDF nor HEK proliferation was significantly affected by the presence of leukocytes in plasma-derived formulations independently of the medical situation of the donor. Considering the effect of leukocyte inclusion, cell-based assays revealed that HDF migration rates were significantly lower in LSP-supplemented cultures than in their SP-supplemented counterparts regardless of the health status of the donors (pH = 0.0015, pAD = 0.041, pPS = 0.0001, and pLS ≤ 0.0001). By the results obtained from the HDF assay, decreased HEK motility was observed following cell supplementation with leukocyte-rich supernatants extracted from both healthy (*P *= 0.00010) and pathological donors (pPS ≤ 0.0024 and pLS ≤ 0.00010), with the only exception of supernatants obtained from individuals with AD.

Effect of heat inactivation (Immunosafe)

In terms of cell viability, significant differences have been detected between SP and IS-SP obtained from healthy donors (*P *= 0.015). Nevertheless, pairwise comparisons revealed that HEK proliferation increased following supplementation with IS-SP supernatants obtained from pathological donors when compared to their LSP (pAD = 0.00070) or SP counterparts (pPS = 0.024 and pLS = 0.0004). Similarly, the Immunosafe treatment significantly increased cell migration in HDF supplemented with LSP obtained with donors with AD (*P *= 0.012). The effect of heat inactivation on HEK migration was also significant when LSP from donors with LS was used as a cell culture media supplement (*P *= 0.00040).

As depicted in Table 2, when compared to their healthy counterparts, no significant differences were detected for ROS levels in HDFs or HEKs supplemented with plasma-derived formulations obtained from donors with chronic skin disorders. After heat inactivation, oxidative stress decreased significantly in HEKs supplemented with IS-LSP from individuals with AD (*P *≤ 0.0001) and LS (*P *= 0.012). Moreover, considering plasma-derived formulations from donors with PS, ROS levels dropped significantly after leukocyte inclusion in HDFs (*P *= 0.020) and HEKs (*P *= 0.036).

**Table 2:** Intracellular reactive oxygen species (ROS) of inflamed in vitro models (arbitrary units) supplemented with plasma-derived formulations obtained from healthy and pathological donors using the following cell lines: (A) HDF and (B) HEK. Symbols denote statistical significance (*P *≤ 0.05): *, differences between pathological donors and healthy donors within a specific formulation; #, differences between raw formulations (SP) and those subjected to the Immunosafe thermal inactivation treatment (IS-SP); and $, differences between PRGF-derived supernatants (SP) and leukocyte-rich supernatants (LSP). HDF, human dermal fibroblast; HEK, human epidermal keratinocyte

Formulation	ROS/cell (arbitrary units)							
	Health		Atopic dermatitis		Psoriasis		Lichen sclerosus	
	HDF	HEK	HDF	HEK	HDF	HEK	HDF	HEK
SP	0.078 (0.073-0.084)	0.054 (0.045-0.063)	0.077 (0.067-0.086)	0.062 (0.049-0.074)	0.080 (0.073-0.088)	0.069 (0.041-0.096)	0.080 (0.069-0.090)	0.065 (0.053-0.077)
IS-SP	0.074 (0.068-0.081)	0.050 (0.039-0.061)	0.076 (0.065-0.087)	0.036 (0.026-0.046)	0.077 (0.066-0.088)	0.057 (0.036-0.078)	0.079 (0.072-0.087)	0.044 (0.033-0.056)
LSP	0.079 (0.074-0.084)	0.049 (0.038-0.060)	0.081 (0.067-0.094)	0.071 (0.046-0.096)	0.064 (0.049-0.080)$	0.052 (0.035-0.068)$	0.083 (0.073-0.092)	0.055 (0.049-0.061)
IS-LSP	0.068 (0.058-0.077)	0.045 (0.036-0.053)	0.069 (0.062-0.076)	0.035 (0.032-0.039)#	0.070 (0.065-0.074)	0.053 (0.038-0.068)	0.079 (0.069-0.088)	0.051 (0.043-0.059)#

## Discussion

During the last decade, the potential effectiveness of PRP-based therapies - either alone or in combination with other drugs, treatments, biomaterials, or stem cells - for managing chronic skin inflammation has been increasingly explored, and some of these investigations have yielded promising results. Vafaei-Nodeh and Kabiri-Abyaneh [28] concluded that long-term management of AD can be effectively achieved using PRP therapy. Accordingly, Yosef et al. [19] highlighted that combining PRP with narrowband ultraviolet B (NB-UVB) can be regarded as a straightforward, acceptable, reliable, and cost-effective approach for managing AD. Following the treatment of 40 patients with inflammatory skin disorders with PRP, Kauhl et al. [18] detected a significant reduction in average lesion size and 50% of all patients achieved complete remission after 53 months. As reviewed by Zaki et al. [29], similar results have been obtained in several studies either using PRP as a monotherapy or as an adjuvant therapy. On the other hand, Chakravdhanula et al. [30] observed that psoriatic patients treated with a combined therapy of PRP and methotrexate (MTX) showed a significant reduction in erythema, induration, and desquamation at each visit, with PS remission achieved after 16 weeks. Comparable results have been observed with PRP used as a monotherapy for treating plaque PS [31]. According to Franic et al. [32], Goldstein et al. [33,34], and Medina Garrido et al. [35], PRP may be also effective for symptom relief in certain patients with LS who do not respond to first-line therapy. In the same line, Casabona et al. [36,37] and Villalpando et al. [38] indicated that PRP can be used as symptomatic treatment of LS due to its capacity to reduce inflammation and improve clinical conditions in women with vulvar LS without the potential side effects derived from immunomodulatory therapies. In addition, the combination therapy of PRP and adipose-derived stem cell (ADSC) has demonstrated significant improvements in LS-related symptoms and hence offers a synergistic approach to address the complex pathophysiology of LS [39]. Nevertheless, the degree of evidence is limited by heterogeneity in PRP preparation and administration protocols.

Due to its strong regenerative properties, PRGF is a standardized biological therapy widely used in multiple medical fields [40]. This versatile biomedical technology is characterized by a moderated enrichment platelet enrichment and leukocyte exclusion [41]. In vitro scientific evidence suggests that high leukocyte contents in PRP can promote the expression of catabolic cascades and inflammatory cytokines [42]. Beneficial biological effects of PRGF on different tissues are mainly derived from a pool of growth factors, including platelet-derived growth factor-AB (PDGF-AB), vascular endothelial growth factor (VEGF), epidermal growth factor (EGF), TGF-β1, insulin-like growth factor (IGF), fibroblast growth factor (FGF), or hepatocyte growth factor (HGF) [43]. As suggested by a preliminary study, PRGF therapy could be considered a promising approach for treating inflammatory skin conditions, since PRGF-based formulations significantly increased tissue viability and reduced the excessive free radical accumulation and cytokine production, such as TNF-α and IL-1, in ex vivo human skin models [44]. Case reports also showed a positive response after PRGF application in terms of skin quality improvement, local erythema decrease, and burning and itching amelioration [45]. However, compositional variations affecting multiple proteins related to key biological processes have been identified recently in PRGF samples obtained from donors with chronic inflammatory skin conditions. As revealed by proteomic profiling, the observed differential abundance patterns were mainly related to complement activation, blood coagulation, and glycolysis and gluconeogenesis-related processes [27].

Consistent with proteomic findings, the results from the present study confirm that the response of inflamed human skin cell cultures to PRGF supplementation can be influenced by the inflammatory status of the blood donor. This observation suggests that the immunogenic potential of plasma is highly dependent on the health condition of the donor, and interindividual variations affecting the inflammatory status could exert a significant impact on PRGF bioactivity. In particular, the observed differences in cell viability, proliferation, and migration rates between healthy-derived PRGF formulations and their counterparts derived from pathological donors affect mainly inflamed HEK cultures. Previous studies have also concluded that the molecular composition of PRP, as affected by the age [46], sex [47], physical activity habits of the donors [48], and individual’s immune profile [19], may condition the magnitude of the biological response. As suggested by cell-based assays, the influence of skin health status on in vitro viability and proliferation of PRGF-derived formulations was higher for HEK. It should be noted that keratinocytes play a crucial structural role in the healing process by covering dermal and mucosal wound surfaces to restore an epithelial barrier with the external environment, but also exert important immune functions [49]. Since white blood cells are the primary source of inflammatory cytokines, such as IL-1 and TNF-α, leukocyte inclusion in PRGF formulations significantly decreased HDF and HEK migration rates [50]. Moreover, enhanced inflamed HEK proliferation and inflamed HDF/HEK migration behavior were observed after supplementing cell cultures with Immunosafe-treated PRGF-derived formulations, particularly leukocyte-rich supernatants. The efficacy of heat-inactivation in reducing the content of immunoglobulin E (IgE) and the complement activity while preserving the content of most of the proteins and morphogens involved in wound healing and tissue regeneration has been highlighted extensively [22,27,44].

Despite the recent publication of a study evaluating the effect of the inflammatory status of donors and different protocol variations on PRGF bioactivity [26], the novelty of the present research relies on the use of inflamed cell models corresponding to predominant cell types in the skin to mimic the response of pathological tissues to supplementation with different PRGF-derived formulations. This study has two key methodological limitations: the small number of participants involved and a significant variability in medication use patterns. In this context, a limited sample size may affect statistical power and hinder reproducibility. Additionally, the presence of confounders in diverse population groups raises concerns, particularly regarding the potential effects of biological medications or anti-inflammatory drugs utilized in the treatment of chronic inflammatory skin conditions on PRGF composition and bioactivity. It should be noted that this preliminary investigation was designed to explore the impact of skin inflammation status and the inclusion of an additional heat-inactivation step on PRGF therapeutic potential, rather than to quantify the overall efficacy within a broader population. Moreover, the statistical methods applied were selected to ensure valid conclusions and to mitigate the impact of the sample size limitation. Future research should focus on assessing how varying health conditions and PRGF preparation protocols influence the composition of PRGF formulations and their biological effects. This should include larger sample sizes and emphasize the control of specific confounding variables in the analysis. Future research should focus on examining the influence of different health conditions or PRGF preparation methods on the proteomic profiles of PRGF formulations, using larger sample sizes and controlling for specific confounding variables.

## Conclusions

This preliminary study suggests that PRGF supernatant may exert beneficial effects on inflamed HEK and HDF bioactivity. However, a significant effect of soluble autoimmune components contained in plasma from individuals with inflammatory skin disorders on cell biological activity has been identified. Consequently, safety measures, including heat inactivation (Immunosafe treatment) or leukocyte exclusion may reduce local side effects in the context of clinical application. It can be concluded that PRGF-based therapy could be regarded as an autologous and versatile approach to promoting tissue regeneration in patients with chronic skin pathologies. In the context of personalized medicine, the composition and bioactivity of PRGF-derived formulations can be modified by implementing additional treatments that can be used to design specific medical needs.
